# Short relaxation times but long transient times in both simple and complex reaction networks

**DOI:** 10.1098/rsif.2016.0388

**Published:** 2016-07

**Authors:** Adrien Henry, Olivier C. Martin

**Affiliations:** GQE-Le Moulon, INRA, Univ. Paris-Sud, CNRS, AgroParisTech, Université Paris-Saclay, 91190 Gif-sur-Yvette, France

**Keywords:** characteristic times, reaction networks, tracers, biomodels

## Abstract

When relaxation towards an equilibrium or steady state is exponential at large times, one usually considers that the associated relaxation time *τ*, i.e. the inverse of the decay rate, is the longest characteristic time in the system. However, that need not be true, other times such as the *lifetime* of an infinitesimal perturbation can be much longer. In the present work, we demonstrate that this paradoxical property can arise even in quite simple systems such as a linear chain of reactions obeying mass action (MA) kinetics. By mathematical analysis of simple reaction networks, we pin-point the reason why the standard relaxation time does not provide relevant information on the potentially long transient times of typical infinitesimal perturbations. Overall, we consider four characteristic times and study their behaviour in both simple linear chains and in more complex reaction networks taken from the publicly available database ‘Biomodels’. In all these systems, whether involving MA rates, Michaelis–Menten reversible kinetics, or phenomenological laws for reaction rates, we find that the characteristic times corresponding to lifetimes of tracers and of concentration perturbations can be significantly longer than *τ*.

## 1. Introduction

Networks have been used to model systems involving large numbers of components, agents or species [1]. Of particular interest are the effects arising in such systems either because of out-of-equilibrium dynamics or through equilibrium phase transitions. Collective effects are generally associated with slow dynamics, i.e. characteristic times that are much larger than the microscopic times associated with elementary processes. In the present work, our focus is on the emergence of large characteristic times in *reaction* networks close to their steady state. There are many ways to define a characteristic time in a dynamical system. The simplest is via the asymptotic relaxation towards the steady state [2,3], relaxation which often will be exponential. If so, the amplitude of the perturbation or ‘distance’ to the steady state will decay as exp(−*t*/*τ*) at very long times, from which one then defines *τ* to be the *relaxation time*. Because all eigenmodes of the linearized equations decay on a timescale shorter or equal to *τ*, it is common practice to assume that *τ* is the longest characteristic timescale in the system. As a consequence, one usually takes for granted the rule of thumb that the steady states will pretty much be reached within two or three times *τ* and that any study can focus on just determining *τ* [4]. In metabolic networks, this rule of thumb is often used to classify different timescales [3,5,6]. However that assumption is not always correct and refinements are sometimes necessary when considering arbitrary characteristic times. Our goal here is to investigate cases where much larger times can arise. The present study focuses on reaction networks for specificity, but our framework is more generally applicable to any system.

Reaction networks involve species that can transform one into another. If the species are molecular, one can get insights into the dynamics of the system by introducing an isotopic *tracer* and by following in time its incorporation into the different molecular species [7]. Assume that the reaction network is in contact with outside reservoirs, and let *t*_t_ be the time the tracer takes to exit the system. Surprisingly, the maximum of *t*_t_, corresponding to the tracer's lifetime in the system [8–10] (and sometimes called the mean residence time of the tracer), can be much *greater* than *τ*. The object of our work is to understand such a possibility, pointing in particular to the danger of assuming that *τ* is the main and longest characteristic time in these systems. For pedagogical reasons, we will begin by treating one-dimensional networks because an in-depth analytical treatment is feasible there, from which one can easily understand the influence of network size. We will then study more general systems using reaction networks published by other authors. In all cases, we compare the behaviours of *four* characteristic times in these systems, investigating the causes that can render them non-informative or make their ratios diverge.

## 2. Model and methods

### 2.1. Reaction networks and our one-dimensional kinetic models

A metabolic network consists of a set of reactions and associated metabolites. It is convenient to represent such a network as a graph where the nodes are associated with metabolites; these are linked together by edges when there is a reaction that includes them as substrate and product. Such edges may be uni- or bi-directional, accounting for the reversibility of the associated reaction. Let there be *N* metabolites *M_i_* (*i* = 1, … , *N*) and define *C_i_* as the concentration of *M_i_*. We are interested in the dynamics of the *C_i_*, i.e. how these quantities change with time and in the corresponding fluxes through the different reactions. Specifically, we shall study the dynamics close to the system's steady state and we shall probe the associated characteristic times. To facilitate the mathematical understanding of these times, we shall first focus on a particular kind of network consisting of a linear chain of reactions. In that situation, we order the metabolites from 0 to *N* + 1 where the metabolite *M_i_* is the product of reaction *R_i_* whose substrate is metabolite *M_i_*_− 1_2.1
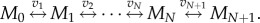


The metabolites *M*_0_ and *M_N_*_+ 1_ reside in infinite reservoirs at the two extremities of the linear chain so their concentrations are constant. By convention, the forward direction in such a chain goes from *M*_0_ to *M_N_*_+ 1_. Once the characteristic times in this system are understood, we shall use the insight thereby gained to probe the situation in more realistic metabolic networks with branches and loops.

Reactions transform metabolites into other metabolites but it is still necessary to specify the actual kinetics. When a reaction happens spontaneously, without the need for a catalyst, it can be modelled by a mass action (MA) rate law where the net flux is given by2.2
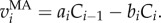
To be specific, one can consider using the standard convention whereby concentrations are measured in moles per litre and fluxes in moles per litre per second. The parameter *a_i_* (respectively, *b_i_*) is then the probability per second that a molecule of metabolite *M_i_*_− 1_ (respectively, *M_i_*) spontaneously transforms into a molecule of metabolite *M_i_* (respectively, *M_i_*_− 1_). Note that equation (2.2) gives the total flux which is the forward flux minus the backward flux.

In practice, one is often interested in catalysed reactions where the spontaneous rates are terribly low. For instance, in biochemistry, most reactions are catalysed by enzymes; catalysis can lead to enhancement of rates by a factor of 10^10^ or more. For any such enzymatic reaction, the rate may be limited by the amount of enzyme and is no longer entirely proportional to metabolite concentration. Generally, the relationship between substrate concentration and reaction rate grows linearly at low concentrations and then saturates at high concentrations of substrate. The reaction kinetics in this situation are typically modelled by the so-called reversible Michaelis–Menten–Henri (MMH) Law [11]. In the case of a reaction involving one substrate and one product, the flux is given by2.3
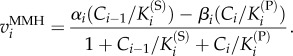


Here, *α_i_* is the maximum rate in the forward direction, reached when the substrate is in large excess and the product is absent. Similarly, *β_i_* is the maximum rate in the backward direction. The maximum forward rate is proportional to the enzyme concentration and is often decomposed as *α* = *k*_cat_*E* with *E* being the enzyme concentration and *k*_cat_ the maximum number of reactions catalysed by one molecule of enzyme per unit of time. 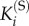 and 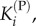 called the Michaelis constants, respectively for substrate and product, are characteristic concentrations which set the scale for when the reaction becomes saturated in substrate or in product. For an MMH reaction in the absence of the product, *K*^(S)^ is the concentration for which the rate is at half of its maximum value.

### 2.2. Determining steady states

When a physical system is not driven by outside forces, it goes to its equilibrium state where all net reaction fluxes are 0. In the context of our one-dimensional model, that can only arise if the free energies of the two reservoirs are equal, corresponding to tuning the concentrations so that their ratio is the equilibrium one. Outside of that special case, the system will be out of equilibrium and concentrations will change in time until a steady state is reached which necessarily will have non-zero fluxes. This steady state is generally unique if there are no regulatory processes but for our study to be completely general, we will not assume uniqueness of the steady state, we shall simply consider a stable steady state and investigate its characteristic times.

To determine a steady state, we solve numerically the set of steady-state equations d*C_i_*/d*t* = 0. (We use the root finding routine ‘find-root’ in Python.) For any set of kinetic parameters or boundary conditions, e.g. the concentrations *C*_0_ and *C_N_*_+ 1_ in the one-dimensional models, this computation leads to a steady-state concentration vector ***C***^ss^. It is necessary to check that the resulting steady state is linearly stable. This check can be performed using the linearized equations about the steady state. If ***δC*** is the (infinitesimal) difference between the actual concentrations and those in the steady state, one has2.4
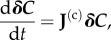
where **J**^(c)^ is the *N* × *N* Jacobian matrix with indices *i* and *j* going from 1 to *N*; the superscript ‘c’ refers to the fact that it describes the (linearized) dynamics of (perturbed) *concentrations*. The steady state is stable if the real part of each eigenvalue of the Jacobian is negative.

For the specific case of our one-dimensional models, one has2.5
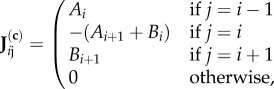
where the *A_i_* and *B_i_* are related to the terms entering equation (2.2) for MA and equation (2.3) for MMH as specified in table 1.

**Table 1.** Value of the *A* and *B* parameters for the four situations considered. *F* and 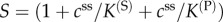 are, respectively, the flux and a measure of flux saturation in the steady state, the system being by hypothesis homogeneous. The ‘c’ (respectively the ‘t’) appended to MA and MMH denotes perturbed concentrations (respectively, tracer concentrations).

parameter	MA*-c*	MA*-t*	MMH*-c*	MMH*-t*
*A*	*a*	*a*	(*α* − *F*)/*K*^(S)^*S*	*α*/*K*^(S)^*S*
*B*	*b*	*b*	(*β* + *F*)/*K*^(P)^*S*	*β*/*K*^(P)^*S*

In all the parametrized models we examined, we found a single steady state and it was stable, so hereafter all eigenvalues of **J**^(**c**)^ will implicitly be assumed to have a negative value for their real part.

### 2.3. Defining four characteristic times

*τ*_c_: The first characteristic time is the *relaxation* time defined as 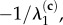 where 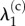 is the real part of the leading eigenvalue of **J**^(c)^ having the largest real part (closest to 0 from the negative side). Because this time is defined via the linearized dynamics for the *concentrations* about the steady state, we shall refer to it as *τ*_c_.

**T**_t_: The second characteristic time is the previously mentioned tracer *lifetime* (or mean residence time), which we denote by *T*_t_. The motivation for introducing this quantity comes from tracer experiments in chemical networks where isotopic labels are used to follow atoms as reactions progress. Instead of introducing a perturbation to concentrations, this approach labels atoms of one metabolite *M_k_* at *t* = 0 without changing any concentrations. In practice, this labelling affects only a fraction of the molecules. The effect of this labelling is also to leave the fluxes unperturbed. The system stays in its steady state, it is just that some of these molecules become labelled. Note that when a labelled metabolite is tranformed into another, tracers follow via the labelled atoms. (See also the electronic supplementary material.)

Let us study the time evolution of the concentrations of these tracers 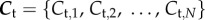 (the subscript ‘t’ is for *tracer*). Let 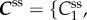 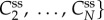 be the steady-state concentrations of the metabolites (labelled or not). Consider the reaction *R_i_* and let 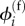 be its forward flux and 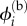 its backward flux in the steady state. Then the change in the labelled concentration *C*_t,*i*_ will include an incoming term given by the rescaled forward flux 
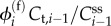
 because all metabolite molecules (labelled or not) have an equal probability of participating in the reaction *R_i_*. As a result, the dynamics of the tracer concentrations is2.6
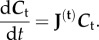


In the specific case of the one-dimensional models, we have2.7
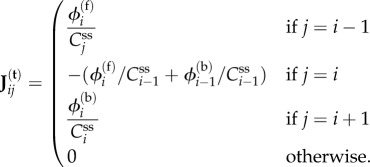
Note that these linear dynamics are exact even if *C*_t,*i*_ is not infinitesimal. In general, the matrix **J**^(t)^ has no reason to be identical to **J**^(**c**)^. By exponentiating, one has the expression for the labelled concentrations at all times: 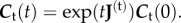 The lifetime of the tracer is then defined to be the integral over time of the proportion of the labelled atoms that are still present in the system. This quantity depends on the site at which the tracer is initially introduced. We thus define the tracer lifetime *T*_t_ as the largest such time when considering all possible initial sites2.8
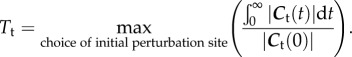


In the above equation, |***C***_t_(*t*)| is the norm of the corresponding vector. For our study, we use the *L*_1_ norm (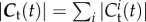) because it makes more sense for an atomic tracer which is conserved until it is pushed out of the system. Note also that *T*_t_ in equation (2.8) is the direct analogue of the mean lifetime of a decaying positive *scalar* quantity; the norm allows one to extend the notion to a vector in a straightforward manner.

**T**_c_: The previous definition of lifetime of a tracer can be generalized to the lifetime of any quantity and in particular to a perturbation applied to steady-state concentrations. Suppose one introduces at *t* = 0 an infinitesimal perturbation in the concentrations, ***δC***(0). In what follows, and just as for tracers, this vector at *t* = 0 will have a non-zero component only for a single metabolite of the system. Then according to equation (2.5), 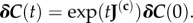 In direct analogy with equation (2.8), we define the concentration lifetime *T*_c_ as2.9
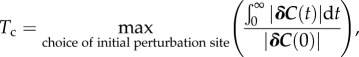
providing a third characteristic time of our system, referred to as the lifetime of a concentration perturbation. To be completely general, both here and for the tracer lifetimes, the vectors of concentrations should be taken as the deviations of their values from their long time limit. Indeed, if there were no reservoir and thus no exit possible of the atoms, the long time limit of the perturbation or tracer concentration would not be 0.

*τ*_**t**_: Our fourth and last characteristic time is *τ*_t_, defined as 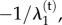 where 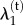 is here the real part of the leading eigenvalue of **J**^(**t**)^. It corresponds thus to the usual relaxation time but for the tracer molecules rather than for the metabolite concentrations, thus the subscript ‘t’.

## 3. Behaviour of characteristic times in the one-dimensional models

As can be seen from the four characteristic times defined in the previous section, we distinguish two aspects of a metabolic system: (i) the dynamics of an infinitesimal perturbation in the concentration of metabolites and (ii) the spreading and drift of tracers. Both aspects can be considered, whether the reaction kinetics are given by MA or by MMH rate laws. In each case, one can define both the standard relaxation time based on the asymptotic decay rate and a lifetime which measures the characteristic time needed for the system to return close to its steady state. In the case of a linear chain of reactions with the same kinetic parameters, that homogeneity allows us to obtain some results analytically. For instance, in the case of MA, the linearized dynamics (given in terms of **J**^(**t**)^ and **J**^(**c**)^) are independent of the steady state considered. Thus, the characteristic times will be independent of the sign and intensity of the flux going through the network: the concentrations of *M*_0_ and *M_N_*_+ 1_ are irrelevant! Furthermore, the Jacobian matrices are sufficiently simple for one to obtain in closed form the eigenvectors and eigenvalues. In the case of an MMH framework, when one performs the linearization about the steady state, the resulting system is homogeneous only if the steady state itself is homogeneous, which requires that all the metabolites have the same concentrations. When this is the case, we again obtain the steady state in closed form. And just as for MA, the eigenvectors and eigenvalues can be derived analytically, which gives us then the formulae for *τ*_c_ and *τ*_t_. Unfortunately, for both MA and MMH, the study of the lifetimes *T*_t_ and *T*_c_ requires resorting to numerical methods to exploit equations (2.8) and (2.9) (see the electronic supplementary material for details). Nevertheless, these algorithms are relatively straightforward as they reduce to calculating exponentials of the matrices **J**^(t)^ and **J**^(**c**)^ and performing the integrations in equations (2.8) and (2.9). For the initial perturbation, for simplicity we take ***δC***(0) and ***C***_t_(0) to vanish everywhere except on one site where it is set to 1. It results in *N* possibilities to measure the lifetime, to remove the dependence on the site we choose to define *T*_t_ or *T*_c_ as the maximum over the *N* possibilities.

### 3.1. Long transient times drive the gap between lifetimes and relaxation times

**Figure 1.**
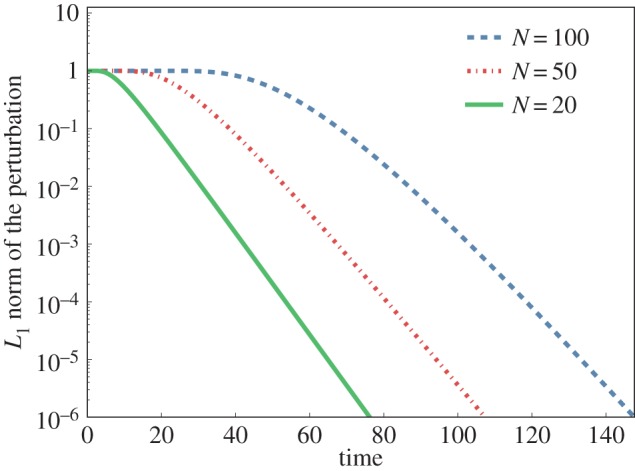
Decrease with time of |***C***_t_|, the *L*_1_ norm of the vector of concentrations of a tracer. Identical results apply to |***δC***|, the *L*_1_ norm of the vector of perturbed concentrations. The initial perturbation at *t* = 0 is localized at a site in the middle of the chain of reactions. The *y*-axis is on a log scale so that one can see the asymptotic exponential decay as a straight line of slope −1/*τ*: *τ*_20_ = 4.92, *τ*_50_ = 5.65 and *τ*_100_ = 5.78. All *N* MA reactions have *a* = 2 and *b* = 1. Cases with *N* = 20, 50 and 100 are shown.

The integral in equation (2.8) depends on 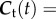 
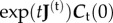
 which can be written using spectral decomposition as a sum of *N* terms, each term being associated with one eigenmode and having the time dependence 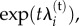 where 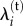 is the associated eigenvalue. When *N* = 1, ***C***_t_(*t*) is a constant times a single decaying exponential. Plugging into equation (2.8) then reveals that *T*_t_ = *τ*_t_. The paradox whereby *T*_t_ can be much larger than *τ*_t_ arises when 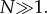 It is true that each of the *N* terms contributing to the spectral decomposition of ***C***_t_(*t*) decays in magnitude at least as fast as exp(−*t*/*τ*) but that does *not* mean that the sum of these terms has that behaviour on timescales comparable to *τ*. Indeed, the terms are not all of the same sign, and their cancellations can lead to long transients before the asymptotic behaviour (the exponential decay) prevails. To illustrate this, we show in figure 1 the *L*_1_ norm of ***C***_t_(*t*) as a function of *t* in our toy model consisting of a linear chain with *a*'s and *b*'s identical across MA reactions. At large times, one sees the exponential decay (a straight line on this semi-log plot) but this asymptotic behaviour may set in at times only much longer than *τ* itself. The cancellation at short times just mentioned is particularly striking: the curve is very flat for a very long time before it begins to decrease. That waiting time contributes to the large difference between *T*_t_ and *τ*_t_: one must wait for tracer molecules to be transported through the system. Note that the property of having a very flat curve at initial times is due to the conservation of particles within the system, justifying our use of the *L*_1_ norm instead of the *L*_2_ norm.

### 3.2. Dependence of the characteristic times on *N*

Let us begin with the relaxation times *τ*_t_ and *τ*_c_. Assuming the reactions to all have the same kinetic parameters and that the steady state is also homogeneous (cf. previous remarks), the relaxation time (be it *τ*_c_ or *τ*_t_) can be obtained by using the translation invariance of **J**^(**c**)^ and **J**^(**t**)^. Each eigenvector is a product of a sine and an exponential (see the electronic supplementary material). The formula for the eigenvalues leads to3.1
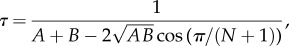
where the quantities *A* and *B* are, respectively, the forward and backward probability of transition per unit time in the equations linearized about the steady state, entering in **J**^(**c**)^ for *τ*_c_ and in **J**^(**t**)^ for *τ*_t_. They depend on whether one considers MA or MMH reaction kinetics and whether one considers a concentration perturbation or a tracer, the different cases being enumerated in table 1.

**Figure 2.**
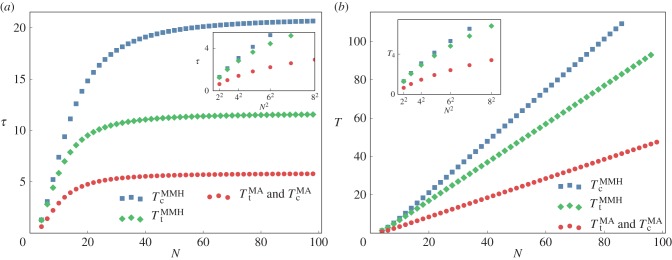
(*a*) Relaxation times (*τ*_t_ and *τ*_c_) and (*b*) lifetimes (*T*_t_ and *T*_c_) for networks between 2 and 100 metabolites long using the MA or the MMH frameworks. Parameters: *a* = 2 and *b* = 1, *K*^(S)^ = *K*^(P)^ = 2 and *α* = *aK*^(S)^ and *β* = *bK*^(P)^ so that the conditions are comparable. The large *N* relaxation times are, respectively, 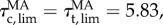 
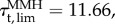
 
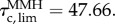
 The crossover sizes between a quadratic and constant or linear behaviour are 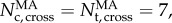 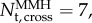 
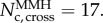
 The insets illustrate the quadratic dependence on *N* for 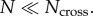

**Figure 3.**
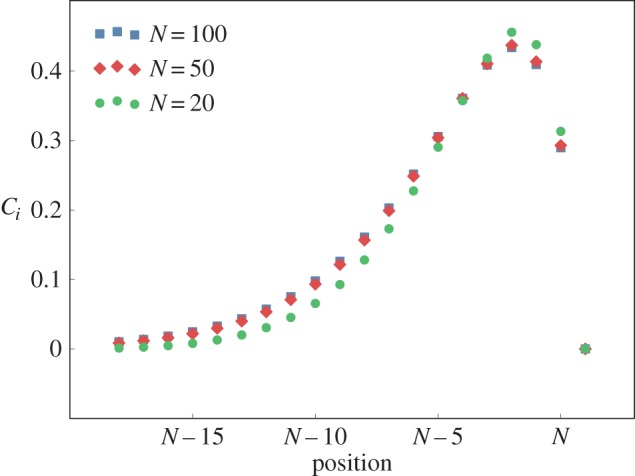
Leading eigenmode profile for the last 20 metabolites of the one-dimensional network when using MA kinetics. Parameters: *a* = 2, *b* = 1, and *N* = 20, 50, 100.

The values of *τ*_t_ and *τ*_c_ in the MA and MMH cases are given by a standardized formula (equation (3.1)), it is just that the proper *A* and *B* coefficients must be used. To understand qualitatively the behaviour of these relaxation times, note first that for MA kinetics, **J**^(**t**)^ = **J**^(**c**)^ so *τ*_t_ = *τ*_c_. Furthermore, consider fixing (*A* + *B*)/2 to a value *Z* and letting *A* − *B* go to 0 in equation (3.1). It is easy to see in such a limit (this applies of course to both the MA and MMH frameworks) that *τ* exhibits two different regimes, one for small chains and one for long chains. For a short chain, 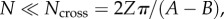 the characteristic times *grow quadratically* with the number *N* of metabolites in the chain, a feature characteristic of diffusing systems for the simple reason that if *A* = *B*, the dynamics is purely diffusive. This is illustrated by the inset of figure 2*a*. By contrast, when *N* is much above this crossover value, *τ*_t_ and *τ*_c_ become independent of the chain length as can be seen directly by setting to 1 the cosine in equation (3.1). This feature is illustrated in the main part of figure 2*a*. To obtain some physical insight into why this large *N* behaviour arises (and thus without mathematically analysing equation (3.1)), let us examine the leading eigenvector of the Jacobian matrix. Its entries depend exponentially on the index *i* of the node and so its profile is mainly concentrated on a few metabolites (about *N*_cross_) at the end of the network. As illustrated in figure 3, if one increases the number of metabolites, that leading eigenmode just gets shifted, keeping very accurately the same profile when measured from the end of the chain. Because this profile determines the eigenvalue, we conclude that increasing *N* hardly affects this leading eigenvalue which itself determines *τ*. Thus, *τ*_c_ and *τ*_t_ become independent of *N* at large *N*.

One may also justify the hierarchy 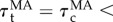 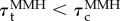 in several limits. First, note that *τ* in equation (3.1) scales with the measure of flux saturation *S* when using MMH kinetics (cf. table 1). As a result, if saturation effects become important, 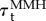 and 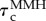 will grow to be much larger than 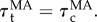 Second, if we consider the expression for *τ* in the limit of small *A* − *B*, we see that *N*_cross_ will be larger when considering perturbations in the concentrations than when considering tracers. As a result, the large *N* limit will set in *later* for perturbations in the concentrations than when following tracers, leading to 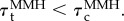


Moving on now to the *T*_c_ and *T*_t_ lifetimes, we found no way to derive closed-form expressions analogous to equation (3.1). Nevertheless, it is possible to understand qualitatively a number of features. In particular, one can again distinguish between two regimes. If *A* − *B* is small, the behaviour for small *N* is diffusion-like again so *T*_c_ and *T*_t_ increase quadratically with *N*. By contrast, for long chains, if *A* ≠ *B*, one has a regime where *T*_c_ and *T*_t_ grow *linearly* with *N*. Arguments similar to the ones presented in the context of the relaxation times *τ* can be invoked to explain these two regimes as follows. In small networks, the diffusion to the two sides of the chain dominates over the mean drift towards one end of the chain. By contrast, within large networks, and assuming *A* > *B*, most of the transient time dominating *T*_t_ and *T*_c_ is dedicated to the transport of the molecules to the *N* + 1 end, therefore that transient time is roughly equal to *N* divided by the drift velocity (which is proportional to (*A* − *B*)). We illustrate these different behaviours in figure 2*b*, where one sees again that the various cases behave similarly with the network length. (We already noted that for MA kinetics, **J**^(**t**)^ = **J**^(**c**)^; as a consequence one has *T*_t_ = *T*_c_ there, just as one has *τ*_t_ = *τ*_c_ in figure 2*a*.)

### 3.3. Effect of the flux saturation on the characteristic times

The major differences between MA and MMH come from the effect of the flux saturation. In the case of the MA rate laws, there is no saturation while saturation effects can be important in MMH kinetics. This difference can lead to much larger characteristic timescales in MMH than in MA whenever the concentrations are larger than *K*^(S)^ or *K*^(P)^. Furthermore, for highly saturated enzymes, the characteristic times can be very different depending on whether one observes a tracer or a perturbation of concentration. Consider a reaction that is near saturation. Introducing a perturbation in the substrate will not greatly change the flux of that reaction and as a result it will take a long time to dissipate the perturbation away. On the other hand, a tracer is essentially unaffected by saturation effects. Indeed, it is not because the reaction is saturated that the tracers cannot participate in the reactions. In effect, the tracers freely pass into reactions that are saturated. The main consequence of this phenomenon is that in MMH *τ*_c_ can be much larger than *τ*_t_ (and *T*_c_ can be much larger than *T*_t_).

**Figure 4.**
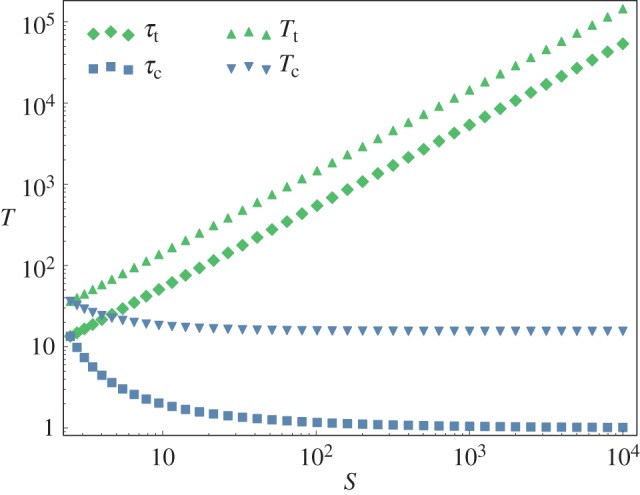
Relaxation times and lifetimes as a function of the measure of flux saturation *S* when using MMH kinetics. Parameters: *α* = 4, *β* = 2, *K*^(P)^ = 2, *N* = 30. To vary the saturation effects, the parameter *K*^(S)^ is changed over a range going from 1 to 10^−4^.

To investigate quantitatively this phenomenon of particular relevance when interpreting kinetic properties from tracer measurements, let us increase saturation effects by reducing *K*^(S)^. *K*^(P)^ could also have been reduced, but when doing so, the flux in the network may reverse which unnecessarily complicates the analysis. Using the parameters of table 1 in equation (3.1) and taking the limit of small *K*^(S)^ gives the following asymptotic behaviour at small *K*^(S)^ of the two relaxation times associated with a tracer (*τ*_t_) and with a concentration perturbation (*τ*_c_):3.2a
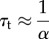
3.2b
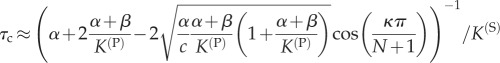
We see from these equations that *τ*_t_ becomes independent of the saturation while *τ*_c_ diverges linearly with 1/*K*^(S)^. Note that the measure of flux saturation *S* = 1 + *c*^ss^/*K*^(S)^ + *c*^ss^/*K*^(P)^ scales in the same way for small *K*^(S)^. In figure 4, we show the dependence of the *τ*s and the *T*s on the saturation *S* for both a tracer and a concentration perturbation, assuming MMH rate laws. Not surprisingly, *T*_c_ is strongly affected by *S*, just as *τ*_c_ is.

## 4. Behaviour of characteristic times in more general metabolic networks

### 4.1. Effects of disorder in a one-dimensional reaction network

**Figure 5.**
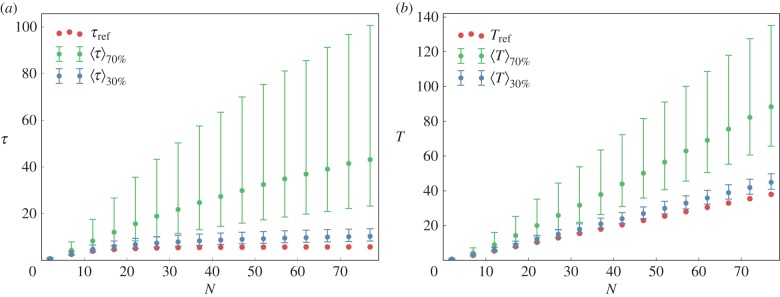
Median relaxation time (*a*) and lifetime (*b*) as a function of *N*. The median of a time is denoted by 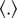 and the subscript in (*b*) of that gives the value used for CV, the coefficient of variation of the reaction rates. Because MA kinetics are used, we have *τ*_t_ = *τ*_c_ (referred to as *τ* in the figure) and similarly *T*_t_ = *T*_c_ (referred to as *T* in the figure). The cases without disorder (CV = 0) do not need any averaging and are referred to as *τ*_ref_ and *T*_ref_. The error bars were obtained by taking one standard deviation on both sides of the median of the distribution. Parameters: *a* = 2, *b* = 1, CV = 30% and 70%.

In the disordered (i.e. heterogeneous) case we now consider, the rates ‘*a*’ and ‘*b*’ for the different reactions are taken to be independent random variables. Because every rate is a positive variable, we draw it from a lognormal distribution, i.e. the natural logarithm of a rate *r_i_* is distributed according to a Gaussian of mean *μ* and standard deviation *σ*. Consequently, the mean of *r_i_* is 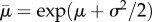 and its variance is 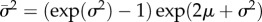. We impose 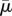 to be equal to the value of the rate in the homogeneous case. An appealing feature of that way of introducing disorder is that the mean drift velocity of a marked molecule in MA remains unchanged, being equal to its disorder average, *E*[*a_i_* − *b_i_*]. We are then left with the parameter 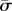 which can go from 0 to ∞ and quantifies the intensity of the disorder. In practice, we use the same coefficient of variation (CV) for the ‘on’ and the ‘off’ reaction rates, corresponding to a single measure of intensity of disorder: 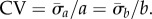


For weak disorder, one expects little change in the values of the characteristic times (*τ*_c_, *τ*_t_, *T*_c_, *T*_t_) compared with the homogeneous case. However, for stronger disorder, the characteristic times typically do increase significantly with CV. To identify the typical behaviour, we have determined these characteristic times for 10 000 realizations of the disorder and calculated the median values. For *τ*_t_ and *τ*_c_, we illustrate our results in figure 5*a* in the case of MA, where those two quantities are equal. The relaxation time increases relatively mildly (cf. the scales) at low CV but increases more markedly when CV becomes larger than 30%. Furthermore, instead of having a limit at large *N* as happens in the absence of disorder, it seems that the presence of disorder makes the relaxation time diverge slowly as *N* → ∞, perhaps logarithmically.

Consider now the effects of disorder on the *lifetimes*. In MA, *T*_t_ = *T*_c_, even in the presence of disorder. We display in figure 5*b* the dependence of these quantities on *N* for several values of CV and see that disorder has little effect as long as CV is small. This can be justified by noticing that the drift velocity of a molecule at site *i* is *a_i_* − *b_i_*_− 1_ and its ensemble average (as in an annealed approximation) is the same as without disorder, namely *a* − *b*. At large disorder, this argument fails because the quenched and annealed averages are very different. A simple way to see this is to consider an extreme case where fluctuations are large. Clearly, a very large value of ‘*a*’ at one site will not compensate a very small value at another site. As expected, at large CV, effects of disorder become very significant. The reason should be clear: *T*_t_ and *T*_c_ are sensitive to unfavourable reactions (for instance, where *a* is small) throughout the whole set of reactions. Finally, just as for relaxation times, it seems that the presence of disorder increases the median values of lifetimes by a factor that diverges slowly (perhaps logarithmically) as *N* → ∞.

### 4.2. Networks with branches and loops

Although quite a few biosynthetic pathways include successive steps forming a linear chain of enzymatic reactions, the one-dimensional systems considered so far remain toy models because in all known organisms, large-scale biochemical metabolic networks have numerous branches and loops. It is thus necessary to consider how characteristic timescales might be affected by such structures. Rather than produce artificial networks including those features, it is more relevant to study directly the various kinetic models of metabolism that have been proposed in the literature. The repository ‘Biomodels’ [12,13] provides the gold standards for such models both because the models must past tests to be deposited and because their availability ensures that they can be compared to state of the art. Focusing further on those models that have been manually curated, we are left with only a handful of cases. The reason for this paucity of models is that measuring kinetic constants of enzymes is a very difficult task so almost always when building a kinetic model the modeller has to use indirect methods to overcome the problem of dealing with many unknown parameters. We studied four of these models, published in [14–17].

For each of those four kinetic models, we first downloaded its SBML specification [13] from the repository and exported the ordinary differential equations into Python code that can be processed. Once in our format, we determined the steady state of the network of reactions and we then computed the matrices **J**^(**t**)^ and **J**^(**c**)^. The associated leading eigenvectors and eigenvalues were obtained using the inverse power method, thereby providing the values of *τ*_t_ and *τ*_c_. Furthermore, numerical integration was used to compute *T*_t_ and *T*_c_ according to equations (2.8) and (2.9), but see also the electronic supplementary material for additional details.

The initial perturbation was taken to be localized on any metabolite of the network. The global lifetime is chosen as the maximum these perturbation lifetimes.

In table 2, we provide the values of the four characteristic times for each of the Biomodels studied. The first model [14] contains the reactions for glycolysis in *Saccharomyces cerevisiae* (baker's yeast). It has 17 reactions, mostly of the reversible MMH type, and there are 14 internal metabolites. Glucose is an external metabolite which enters the metabolism and then gets transformed. A total of three compounds can be excreted, all irreversibly. The characteristic times of this model are modest, from a few seconds to a few minutes. Further inspection shows that the ordering of these four values follows the same pattern as in our one-dimensional toy model, namely4.1
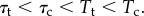
These inequalities can be motivated as follows. First, we expect *τ*_t_ < *τ*_c_ and *T*_t_ < *T*_c_ whenever there are Michaelis–Menten reactions subject to saturation effects. Indeed, the saturation of flux in a reaction can prevent a concentration fluctuation from being evacuated but it will not prevent labelled atoms from going through (i.e. from participating to the flux). Secondly, in our toy model, the *τ*s are relatively insensitive to processes inside the network: they depend mainly on reactions close to the excreted metabolites. By contrast, the *T*s depend on drift throughout the whole network. Thus, if the network is large one can expect the *T*s to be larger than the *τ*s.

**Table 2.** Values of the characteristic times *τ*_c_, *τ*_t_, *T*_c_ and *T*_t_ in seconds for the four manually curated models [14–17] which we have studied. All are available in the Biomodels repository [12].

time (s)	*τ*_t_	*τ*_c_	*T*_t_	*T*_c_
model 1 [14]	3.14	6.21	6.61	46.4
model 2 [15]	11.6	7.54	35.9	11,12
model 3 [16]	2.83 × 10^−2^	5.05	0.109	12.4
model 4 [17]	2715	4.34 × 10^5^	1.18 × 10^4^	9.50 × 10^5^

The other models partly follow this same pattern (cf. table 2). Model 2 contains the reactions for glycolysis and the pentose phosphate pathway in *Escherichia coli* [15]. It has 48 reactions and 17 internal metabolites, but we needed to remove the model's explicit time dependence to allow a steady state. The main difference with model 1 is the organism considered and the glucose steady state uptake rate (3.1 µmol s^−1^ l^−1^ compared to 1.5 mmol s^−1^ l^−1^). The innequalities equation (4.1) are not respected since *τ*_t_ > *τ*_c_ and *T*_t_ > *T*_c_. Model 3 contains the glycolysis and the pentose phosphate pathway, but for a human cancer cell. It has 29 reactions and 34 internal metabolites. The inequalities of equation (4.1) are satisfied except that *τ*_c_ > *T*_t_. This ‘discrepancy’ may be due to the fact that the diameter of the network is modest while saturation effects are important.

Model 4 contains the reactions for the biosynthesis of purines in *E. coli* [17]. It has a total of 29 reactions and 18 internal metabolites. The main difference compared to the other three models is that the formalism uses kinetics that are neither MA nor MMH: the forward and backward rates of the reactions are fractional powers of the concentrations of the metabolites. Such fractional powers are often used phenomenologically to parametrize allosteric or regulatory effects; they have the drawback that the flux may rise very steeply when starting with low concentrations. Although equation (4.1) is qualitatively respected, this model may have further pathologies as suggested by the huge values of all the four characteristic times.

## 5. Discussion and conclusion

In dissipative systems, relaxation is often driven by the local dynamics, and as a result characteristic times of the system (and in particular relaxation times) are comparable to that of the individual processes. However, in systems where atoms or other particles are conserved, the diffusion and drift of the conserved quantities can significantly increase system characteristic times. Pure diffusion provides a simple example of this effect: on a linear lattice of *N* nodes, the characteristic times of the whole system grow as *N*^2^. Our work in this paper focuses on the consequences of drift. For simplicity, we do this in the context of reaction networks operating close to their steady state. If a concentration fluctuation is introduced relative to the steady state, its damping will generally be associated with both a spreading out (diffusion) and also an overall drift. The timescale for evacuating such a perturbation is what we call its lifetime *T* (cf. equations (2.8) and (2.9)), though in other contexts it can be referred to as the mean residence or transit time. In the absence of drift, corresponding to a simple diffusive regime, the lifetime *T* scales as the square of the diameter of the network, a scaling that also arises for the standard measure of the time to return to equilibrium, i.e. the relaxation time *τ*. In most systems, *τ* corresponds in fact the longest characteristic time and there are no further subtleties. However, for almost any reaction network of interest, one has both diffusion and drift. More generally, out of equilibrium systems will have fluxes, and such fluxes may drive conserved particles out of the network. In the presence of such drift, a perturbation's lifetime *T* can scale as the diameter of the network, divided by a characteristic drift velocity which is related to the presence of flux and provides the drift intensity. Interestingly, in this out-of-equilibrium situation, the relaxation time *τ* is no longer informative about the timescale of the (slow) process which evacuates perturbations. In particular, in our toy model consisting of a homogeneous linear chain of reactions, *τ* does not grow with the system size while *T* grows linearly. This can be understood quite intuitively from figure 1 where we see that a perturbation must first drift to the end of the network before the asymptotic behaviour (an exponential decay at the rate 1/*τ*) sets in. *The perturbation's lifetime can thus be much longer than the relaxation time.* We showed analytically how that happened in our one-dimensional homogeneous systems. Indeed, in the presence of drift, the linearized dynamics can be decomposed into eigenvectors, but the leading eigenvector determining *τ* is concentrated near the metabolites that can be excreted. As a result, *τ* is quite insensitive to the size of the network while *T* inevitably increases with network size since the evacuation of a perturbation requires it to cross some fraction of the diameter of the network. When studying the extension to a non-homogeneous system of reactions, it was necessary to take a numerical approach. In our toy one-dimensional network, we introduced heterogeneities into the individual reaction rates. For mild heterogeneities, we find that the effects are small, but as one increases the CV of the reaction rates, the effects of disorder become significantly stronger, increasing in particular the median values of *τ* and of *T*. Interestingly, these heterogeneities drive *τ* to grow with system size as illustrated in figure 5. Empirically, this growth with *N* is slow, perhaps only logarithmic with *N*. Similarly, in the presence of disorder, it seems that *T* grows a bit faster than *N*, perhaps logarithmically faster.

The overall phenomena found are most easily understood when the reactions obey MA, but they arise also for MMH reaction laws. For this last case, the presence of a saturation of the flux with concentration of metabolites exacerbates the difference between characteristic times associated with concentrations versus tracers as is illustrated in figure 4. This is expected because when the network has a bottleneck due to an enzyme becoming saturated, diffusion and drift are ineffective for evacuating an excess concentration while the dynamics of tracers will be barely affected. This feature of tracers means that although they form an irreplaceable tool for measuring fluxes, their naive use can lead one to severely underestimate the longest characteristic times in such reaction networks.

Given any model network with specified dynamics, it is possible to estimate its relaxation times and lifetimes (cf. equations (2.8) and (2.9)) in the context of concentration perturbations or of tracers. The four associated characteristic times will typically follow the inequalities given in equation (4.1) as they do in the one-dimensional models. If a case leads to anomalous results, either because the inequalities are violated or because some of the characteristic times are unreasonable (too short or too long), the reason for the anomaly should be found or the reliability of the model should be questioned. We encountered precisely this situation for the model [17] from the Biomodels database. The authors of that model introduced kinetic laws which reflect a control by some downstream products upon upstream reaction rates, control in the spirit of what is known to arise in several case studies of biosynthetic pathways. Such feedback could possibly lead to having *T*_c_ > *T*_t_ but nevertheless raises a flag as to possible artefacts coming from the peculiar and unorthodox form of the kinetic laws introduced in [17]. The fact that the characteristic times in this model are about a million times larger than expected certainly adds currency to the claim that the model might be defective in some way.

## Supplementary Material

Supplementary informations: Short relaxation times but long transient times in both simple and complex reaction networks

## Data accessibility

The codes used to produce the data in the paper are available as ipython notebooks at https://github.com/adrienhenry/characteristicTimesNetwork.
