# Supplementary material for: Short relaxation times but long transient times in both simple and complex reaction networks
Source: J R Soc Interface. 2016 Jul;13(120):20160388. doi: 10.1098/rsif.2016.0388 (PMC4971225; doi:10.1098/rsif.2016.0388)
Supplement: Supplementary informations: Short relaxation times but long transient times in both simple and complex reaction networks [file rsif20160388supp1.pdf]

Supplementry informations: Short relaxation times but long transient times in both simple and complex reaction networks

Adrien Henry and Olivier C. Martin

INRA, Univ Paris-Sud, CNRS, APT, UMR 0320 / UMR 8120 Génétique Quantitative et Évolution, F-91190 Gif-sur-Yvette, France

May 13, 2016

1 Analytical derivation of the relaxation time

First, let us derive the general solution of the differential equation governing a set of concentrations $x_m(t)$ $(m = 1, ..., N)$:

$$\frac{d\,x_m}{d\,t} = Ax_{m-1} - (A+B)x_m + Bx_{m+1} \tag{1}$$

Separating the dependence on time and position, we set $x_m(t) = T(t)X_m$. Then

$$\frac{T'}{T} = \frac{AX_{m-1} - (A+B)X_m + BX_{m+1}}{X_m} \tag{2}$$

The left hand side is independent of m while the right hand side is independent of t, so both must be equal to the same constant which we denote by $-\lambda$.

The dependence on time is immediate: $T(t) = T(0)\exp(-\lambda t)$.

The equations eq.2 for the X_m correspond to finding an eigenvector of a matrix H of eigenvalue $-\lambda$. The diagonal elements of H are $-(A+B)$ while the off diagonal parts are restricted to nearest neighbors and are again independent of m. Given this translational invariance, the eigenvectors can be taken to be of the form $X_m = X_0 e^{(\gamma \pm j\omega)m}$. Plugging this in, one has

$$-\lambda = Ae^{-(\gamma\pm j\omega)} - (A+B) + Be^{-(\gamma\pm j\omega)}$$

For the studied system, both A and B are real.

Let us start by searching for the real eigen values. We can solve for the real and imaginary parts:

Imaginary part	Real part
$Ae^{-\gamma}\sin(\mp\omega) + Be^{\gamma}\sin(\pm\omega) = 0$	$\lambda = Ae^{-\gamma}\cos(\omega) - (A+B) + Be^{\gamma}\cos(\omega)$
$\gamma = \frac{1}{2}\log\left(\frac{A}{B}\right)$	$\lambda = 2\sqrt{A\,B}\cos(\omega) - (A+B)$

A general solution of eq.2 is a combination of the two complex conjugates X_m^+ and X_m^-:

$$X_m = C^- X_m^- + C^+ X_m^+ = C^- e^{(\gamma - j\omega)m} + C^+ e^{(\gamma + j\omega)m}$$

Whith boundary conditions $X_0 = X_{N+1} = 0$:

$X_0 = 0$

$$\begin{aligned} 0 &= C^- + C^+ \\ C^- &= -C^+ \\ X_m &= Ce^{\gamma m}\sin(\omega m) \end{aligned}$$

$X_{N+1} = 0$

$$\begin{aligned} \sin(\omega(N+1)) &= 0 \\ \omega_\kappa &= \frac{\kappa\pi}{N+1} \quad \kappa \in \{0,1,...,N-1\}. \end{aligned}$$

The search for real eigen values provides us with N real eigen values which is the rank of the matrix H, thus all the eigen values are real.

$$\begin{aligned} x_m^\kappa(t) &= C^\kappa e^{\frac{1}{2}\log(A/B)m}\sin\left(\frac{m\kappa\pi}{N+1}\right)e^{\lambda_\kappa t} \\ &\text{with} \\ \lambda_\kappa &= 2\sqrt{A\,B}\cos\left(\frac{\kappa\pi}{N+1}\right) - (A+B), \quad \kappa \in \boldsymbol{Z^*} \end{aligned} \tag{3}$$

A relaxation time is associated to each λ_κ:

$$\tau_\kappa = \frac{1}{A+B-2\sqrt{A\,B}\cos\left(\frac{\kappa\pi}{N+1}\right)} \tag{4}$$

In practice, one refers to *the* relaxation time as the longest such times, *i.e.*, $\tau = \frac{1}{A+B-2\sqrt{A\,B}\cos\left(\frac{\pi}{N+1}\right)}$.

Case of a symmetric system: For a system that is left-right symmetric ($A = B$), $\tau \approx \frac{(N+1)^2}{A\pi^2}$ for large N which is characteristic of diffusing systems. (Clearly, if $A = B$, the equations describe a diffusing particle that has no bias towards left or right.)

Case of an asymmetric system: If the system is not left-right symmetric ($A \neq B$), at large N the relaxation time become independent of N (and in fact at fixed κ all τ_κ become independent of N:

$$\tau_\kappa \approx -\frac{1}{2\sqrt{AB} - (A+B)}$$

.

A crossover size for N between the two regimes $\tau \propto N^2$ and $\tau = cst$: If the difference between A and B is sufficiently small, the relaxation time will behave as the case $A = B$, that is τ will scale as N^2 until N is large enough for the regime of constant τ to set in. To understand the scale in N where this crossover behavior arises, let us note $A = B + \epsilon$. Then for large N

$$\begin{aligned} 1/\tau &\approx 2B + \epsilon B - 2B\sqrt{1+\epsilon}(1 - \frac{\pi^2}{2(N+1)^2}) \\ 1/\tau &\approx 2B + \epsilon B - 2B(1 + \frac{\epsilon}{2} - \frac{\epsilon^2}{8})(1 - \frac{\pi^2}{2(N+1)^2}) \\ 1/\tau &\approx 2B + \epsilon B - 2B - B\epsilon + \frac{B\epsilon^2}{4} + \frac{B\pi^2}{(N+1)^2} + \frac{B\epsilon\pi^2}{2(N+1)^2} \\ 1/\tau &\approx \frac{B\epsilon^2}{4} + \frac{B\pi^2}{(N+1)^2} \end{aligned}$$

This last equation shows that one has a crossover between the two regimes when

$$\begin{aligned}\frac{B\epsilon^2}{4} &\approx \frac{B\pi^2}{(N+1)^2}\\ \frac{\epsilon}{2} &\approx \frac{\pi}{(N+1)}\end{aligned}$$

The transition between a diffusing regime and a regime independent on the network size N thus occurs for a characteristic size

$$N^{cross} = 1 + \frac{2B\pi}{A-B} \tag{5}$$

2 Generalisation of the concept of tracer to complex reactions

2.1 Kinetic for a tracer in a linear chain

The case of the linear chain provides a ideal case since every reaction has one substrate and one product. In such a case it is relatively easy to derive the reactions for tracers. Let us consider the reaction:

$$A \leftrightarrow B$$

with the kinetic for the conventional direction from left to right:

$$v = \frac{\alpha\frac{a}{K_A^{(S)}} - \beta\frac{b}{K_B^{(P)}}}{1 + \frac{a}{K_A^{(S)}} + \frac{b}{K_B^{(P)}}} = v_+ - v_- \tag{6}$$

$$\text{with} \quad v_+ = \frac{\alpha\frac{a}{K_A^{(S)}}}{1 + \frac{a}{K_A^{(S)}} + \frac{b}{K_B^{(P)}}} \text{ and } v_- = \frac{\beta\frac{b}{K_B^{(P)}}}{1 + \frac{a}{K_A^{(S)}} + \frac{b}{K_B^{(P)}}}$$

Where a and b are the concentrations of A and B and α, β and the Ks are the parameters of the equations. Note that the rate law is a summary of the difference between the forward(v_+) and backward(v_-) fluxes. When tracer molecules are introduced downtream in a chain of reactions, some of them will move against the current although the global flux of the reactions indicate the opposite direction. The correct expression for the tracer rate is

$$v^t = \frac{a^t}{a^{tot}}v_+ - \frac{b^t}{b^{tot}}v_-(\neq v_+ - v_-) \tag{7}$$

The superscript t and tot stand respectively for tracer and total. Interestingly, the eq.7 is still true at steady state, then the dynamics of the tracers depends on their fraction $\frac{a^t}{a^{tot}}$ or $\frac{b^t}{b^{tot}}$ in the network.

2.2 Kinetic for a tracer in a realistic network

In a more complex reaction such as

$$A + B \leftrightarrow C + D \tag{8}$$

the dynamics of the tracer is not as straight forward as for the linear chain. Now a labelled carbon can go from A to C but it can also go from A to D; its fate depends on the chemical mechanism of the reaction. To investigate the dynamics of a tracer in a whole network, one has to determine the exact mechanism of every reaction, this task is tedious and can becomes for large networks. Thus we decided to add a simplification to the dynamics, during a reaction all the

carbon of the tracer substrate are mixed and shuffled with the carbons of the of the non-tracer substrate. It results that every carbon coming from a labelled A can go either to the pool of labelled C or to the pool of labelled D.

Let us exemplify this on the tracer flow from A to C and D:

$$F = \frac{a^t}{a^{SS}} v_+^{SS}$$

a^{SS} and a^t are the total concentration of A at steady state and the tracer concentration of A. The chance for A^t to take part in the reaction is proportional to its fraction. The flow produces C and D in proportions that depends on the number of carbons in each molecules and in the stoichiometry of the reaction. To prevent carbon creation, the following balance has to be satisfied:

$$\frac{\nu_C n_C + \nu_D n_D}{K} = n_A \Rightarrow K = \frac{\nu_C n_C + \nu_D n_D}{n_A}$$

where the νs are the stoichiometric coefficients and the ns the number of carbons contains in one molecule.

Finally one obtains the evolution

$$\frac{da}{dt} = -F \quad \frac{dc}{dt} = \frac{\nu_C n_C}{K} F \quad \frac{dd}{dt} = \frac{\nu_D n_D}{K} F$$

The get the full dynamics obtained by completting the flows $F_{B \to C+D}$, $F_{C \to A+B}$ and $F_{D \to A+B}$.

3 Computing the different characteristic times

Our characteristic times are defined using infinitesimal perturbations of the steady state. Such a choice has the advantage of facilitating the treatment of the time dependence of the perturbation since one can exploit the linearity of the dynamics. Denoting by $\delta\vec{C}$ the infinitesimal vector giving the concentration deviation from the steady state and by $\mathbf{J}$ the jacobian matrix associated with the linearized dynamics, one has:

$$\delta\vec{C} = \vec{C} - \vec{C^{SS}}$$

$\vec{C}$ is the total concentration and $\vec{C^{SS}}$ is the steady state concentration.

$$\begin{aligned} \frac{d\,\delta\vec{C}}{d\,t} &= \mathbf{J}\delta\vec{C} \\ \delta\vec{C}(t) &= e^{\mathbf{J}t}\delta\vec{C}(t=0) \\ |\delta\vec{C}(t)| &= |e^{\mathbf{J}t}\delta\vec{C}(t=0)| \end{aligned}$$

We showed in the first section that the eigenvalues of $\mathbf{J}$ are all real and negative in the case of a homogeneous linear chain as might be expected in a dissipative system.

Our matrix formulation allows us to conveniently define the "lifetime" associated with a perturbation $\delta\vec{C}(t)$ introduced at time $t = 0$ via

$$\begin{aligned} T &= \frac{1}{|\delta\vec{C}(t=0)|} \int_0^\infty |\delta\vec{C}(t)| dt \\ &= \frac{1}{|\delta\vec{C}(t=0)|} \int_0^\infty |e^{\mathbf{J}t}\delta\vec{C}(t=0)| dt \end{aligned}$$

Because of the linearity, the characteristic time T does not depend on the *amplitude* of the initial perturbation, $|C(t \vec{=} 0)|$. In addition, we shall impose the initial perturbation to be localized to

just one site of the metabolic network. In consequence, and without any loss of generality, we can take the initial perturbation to be positive, and then it is easy to see that all of the components of $\vec{C(t)}$ remain positive at all times. using this property, we can interchange the integration and the taking of the norm if we work with the L_1 norm:

$$\begin{aligned} T &= \frac{1}{|\delta\vec{C}(t=0)|}\left|\int_0^\infty e^{\mathbf{J}t}\delta\vec{C}(t=0)dt\right| \\ &= \frac{1}{|\delta\vec{C}(t=0)|}\left|[\mathbf{J}^{-1}e^{\mathbf{J}t}\delta\vec{C}(t=0)]_0^\infty\right| \\ &= \frac{1}{|\delta\vec{C}(t=0)|}|\mathbf{J}^{-1}\delta\vec{C}(t=0)| \end{aligned}$$

The lifetime T as specified is dependent on the position where the perturbation was introduced at $t = 0$. To overcome this drawback, we define the characteristic time of the system as the maximal lifetime when considering all possible positions of the initial perturbation.

Our global algorithm to compute our characteristic time is then as follows:

Data: vector_param: best_vector,conc_ss: concentration at steady state, F: function returning the derivative the system
Result: lifetime of the system
J = linearize F(. . . ,param) near conc_ss;
max_time = 0;
for *i=1 to i=conc_ss* **do**
  $\delta\vec{C}^0 = \{0,\ldots,0\}$;
  $\delta\vec{C}^0[\mathrm{i}] = 1$;
  time = $\frac{1}{|\delta\vec{C}(t=0)|}|\mathbf{J}^{-1}\delta\vec{C}^0|$;
  if *time>max_time* **then**
    max_time = time;
  end
end
return *max_time*;

Algorithm 1: Algorithm to compute the lifetime

Note that that all the previous derivation is done for the lifetime of a concentration perturbation but it remains valid for the lifetime of a tracer when taking the steaty state vector 0 and replacing the perturbation $\delta\vec{C}$ by the concentration of tracer.
